# Small-Pox Decreasing in New York

**Published:** 1882-01

**Authors:** 


Small-Pox Decreasing in New York.—At the present writing small-pox seems to be on the decrease in this city. For the week ending December 3, thirty-three cases were reported, while there were but seventeen for the week ending December 10. This result is due to the very thorough manner in which each new case is followed up by the Board of Health. Whenever a case is reported the patient is immediately removed to the Riverside Hospital on Blackwell's Island, and each occupant in the house in which the case occurred is vaccinated. A physician then visits the house every day for two weeks thereafter, and thus any new outbreak is easily controlled.

In New Jersey where this plan is not pursued the disease is rapidly increasing. The pest-house at Snake Hill was found too small to accommodate all the sufferers, and several tents have been erected for their reception. The Board of Health has petitioned the Board of Finance to make a suitable appropriation for the purpose of combating its spread, but thus for nothing has been done.

The physicians are doing all they can with their limited resources. One day last week Dr. Converse, the County Physician, stood in the hallway of a tenement house in Ravonia avenue and vaccinated nearly three hundred. Six physicians have also volunteered to devote one day each week to this purpose, if the city authorities will furnish the vaccine.

